# A chromosomal investigation of some European Leiodidae (Coleoptera), with particular focus on Spanish subterranean Leptodirini

**DOI:** 10.3897/CompCytogen.v6i2.2575

**Published:** 2012-03-26

**Authors:** Robert B. Angus, David B. Edwards, Carlos G. Luque, Lucia Labrada

**Affiliations:** 1 Department of Entomology, The Natural History Museum, Cromwell Road, London SW7 5BD, UK; 2 School of Biological Sciences, Royal Holloway, University of London, Egham, Surrey TW20 0EX, UK; 3 Gestión del Patrimonio Bioespeleológico. IMPRESS Group TM Consulting S.L.; Grupo de Espeleología e Investigaciones Subterráneas ‹‹Carballo-Raba››; P.O. Box 879; 39080 Santander; Spain

**Keywords:** Chromosomes, Leiodidae, Leptodirini, *Leiodes* Latreille, 1796, *Catops* Paykull, 1798, *Cantabrogeus* Salgado, 2000, *Espanoliella* Georgiev, 1976, *Fresnedaella* Salgado Labrada & Luque, 2011, *Notidocharis* Jeannel, 1956, *Quaestus* Schaufuss, 1861, Cantabria, triploid

## Abstract

Karyotypes are shown for *Leiodes calcarata* (Erichson, 1845), *Catops coracinus* Kellner, 1846, *Cantabrogeus luquei* (Salgado, 1993), *Espanoliella luquei* Salgado & Fresneda, 2005, *Fresnedaella lucius* Salgado, Labrada & Luque, 2011, *Notidocharis uhagoni* (Sharp 1872), *Quaestus (Quaesticulus) pasensis* Salgado, Labrada & Luque, 2010, all of which are shown to have a diploid number of 20 autosomes plus Xy (♂) or XX (♀) sex chromosomes, as well as an as yet undescribed triploid species of the genus *Cantabrogeus* Salgado, 2000. These results are contrasted with published information, all on Leptodirini, which lists 10 species as having diploid numbers of 22 + Xy or XX. It is shown that the higher chromosome number (n = 11 + X or y) previously reported refers exclusively to the more derived Leptodirini (“infraflagellates”) whereas the lower number (n = 10 + X or y) refers to the less derived surface-dwelling forms and the less derived Leptodirini (“supraflagellates”).

## Introduction

Published information on the chromosomes of Leiodidae is very limited and refers exclusively to subterranean species of the tribe Leptodirini (Durand and Juberthie-Jupeau 1980; Alegre and Escolá 1983; Buzila and Marec 2000). In all data on 10 species have been reported, all with the diploid number 24 (22 + Xy (♂), 22 + XX (♀)). Buzila and Marec (2000) considered this to be the basic chromosome number for the family Leiodidae.

However, investigation of two surface-dwelling forms in 2006 by D.B. Edwards as part of a student project supervised by R.B. Angus, revealed the diploid number 20 + Xy in the males studied. This was a surprise and remained a puzzle until, in December 2009 R.B. Angus received a request from I. Ribera (Barcelona) for help in determining the chromosome number of a cave-dwelling leptodirine from the Cantabria region of northern Spain. Despite intensive searching by C.G. Luque and L. Labrada, only females of this species had been found in the cave so that they suspected the species might be parthenogenetic and wondered if it might be triploid. Investigation of this material showed that the species was indeed triploid, with consistent finding of 33 chromosomes per nucleus. However, while the confirmation that this was a triploid parthenogenetic species was very satisfying, it also added to the puzzle over the diploid number for Leiodidae as this species is was a subterranean member of the tribe Leptodirini, from which the diploid number 24 had been consistently reported. It was therefore decided to extend the investigation to some of the bisexual species to see what the diploid number was in species from Cantabria.

The tribe Leptodirini Lacordaire, 1854 (= Bathysciini Reitter 1906) of the family Leiodidae (Newton 1998, Perreau 2000) is the second largest group (after Carabidae) of subterranean Coleoptera. Nearly all Leptodirini, with a few notable exceptions, inhabit caves or deep soil layers in the Mediterranean basin. This area includes the north and east of the Iberian Peninsula, some Mediterranean islands such as Corsica, Sardinia and Sicily, the southern Alps, Italian and Balkan peninsulas, Carpathian Mountains, southern Russia, the Caucasus, Middle East and Iran (Perreau 2000, 2004; Salgado et al. 2008). The monophyletic origin of the tribe’s western Palaearctic core (Leptodirini excl. Platycholeina) is well supported by both morphological (Fresneda et al. 2007) and molecular (Ribera et al. 2010) evidence.

## Material and methods

**Figure 1.**
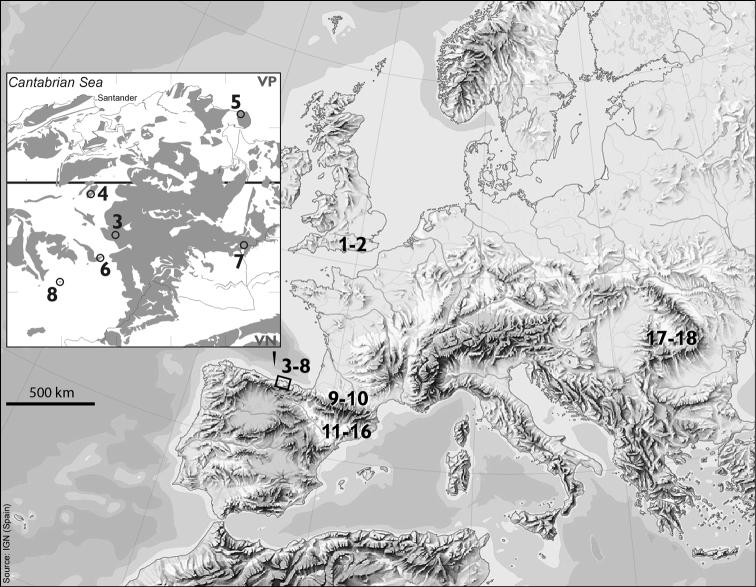
Maps showing the collection sites of the material used in this paper (Nos 1–8), previously published material (Nos 9–18, see Table 2) and areas with carbonate rock outcrops in Cantabria, N Spain (squares 10 × 10 km). See Table 1 for explanation of numbers 1–8, and note that neighbouring sites may share the same number.

The material investigated is listed in Table 1, and the localities from which the material was obtained are marked on the maps shown in Fig. 1. In all cases the number of specimens from which successful preparations have been obtained is given. As these beetles are frequently very small (2 mm or less) the success rate was generally low and preparations were attempted on considerably more specimens than are listed as successful. It should also be noted that there was often considerable mortality in transit, even when material was sent express in cooled containers.

**Table 1.** Material used, localities, map numbers, numbers of specimens giving successful preparations.

**Species**	**Locality with No. on map**	**Number giving successful preparations**
*Leiodes calcarata* (Erichson, 1845)	ENGLAND. Surrey: Virginia Water (No. 1)	1♂
*Catops coracinus* Kellner,1846	ENGLAND. Surrey: Virginia Water (No. 2)	1♂
*Cantabrogeus* sp. (triploid)	SPAIN. Cantabria: Municipal District of San Roque de Riomiera, Covallarco cave (No. 3)	7♀♀
*Cantabrogeus luquei* (Salgado, 1993)	SPAIN. Cantabria: Municipal District of Penagos, Los Gentiles cave (No. 4)	2♂♂, 1♀
*Espanoliella luquei* Salgado & Fresneda, 2005	SPAIN. Cantabria: Municipal District of Santoña, Merino cave (No. 5)	1♂,1♀
*Fresnedaella lucius* Salgado, Labrada & Luque, 2011	SPAIN. Cantabria: Municipal District of Selaya, La Canal de la Cubía cave (No. 6)	1♂,1♀
*Notidocharis uhagoni* (Sharp, 1872)	SPAIN. Cantabria: Municipal District of Ramales, Cullalvera cave-entrance (No. 7)	2♂♂
*Quaestus (Quaesticulus) pasensis* Salgado, Labrada & Luque, 2010	SPAIN. Cantabria: Municipal District of Luena, El Rellano del Mazo cave (No. 8)	2♂♂

The methods of chromosome preparation are as described by Dutton and Angus (2007). The remains of the beetles are either mounted on cards or kept in tubes of 70% ethanol, in the Natural History Museum in London.

For assessment of the chromosomal data in terms of DNA-derived phylogeny we used the dataset from Ribera et al. (2010), plus newly obtained sequences of various Leptodirini species. DNA was extracted from whole specimens with DNeasy Tissue Kits (Quiagen GmbH, Hilden, Germany) in a non-destructive manner to preserve voucher specimens for subsequent morphological study. DNA voucher specimens are deposited in the Institute of Evolutionary Biology (Barcelona, Spain). Seven gene fragments were sequenced: five mitochondrial (3’ end of cytochrome c oxidase subunit 1, *cox1*; an internal fragment of cytochrome b, *cyt b*; and 5’end of large ribosomal unit 16S rDNA plus the Leucine transfer RNA gene plus the 3’ end of NADH dehydrogenase subunit 1, *rrnL+trnL+nad1*, and two nuclear (5’ end of the small ribosomal unit 18S rDNA, *SSU*, and an internal fragment of the large ribosomal unit 28S rDNA, *LSU*). For each fragment both forward and reverse sequences were obtained. Sequences were assembled and edited using Sequencher TM 4.1.4 (Gene Codes, Inc., Ann Arbor, MI). Phylogenetic analysis was conducted using maximum likelihood as implemented in the on-line version of RAxML (which includes an estimation of bootstrap node support, Stamatakis et al. 2008), using GTR+G as the evolutionary model.

## Results

### Surface forms

**Figure 2.**
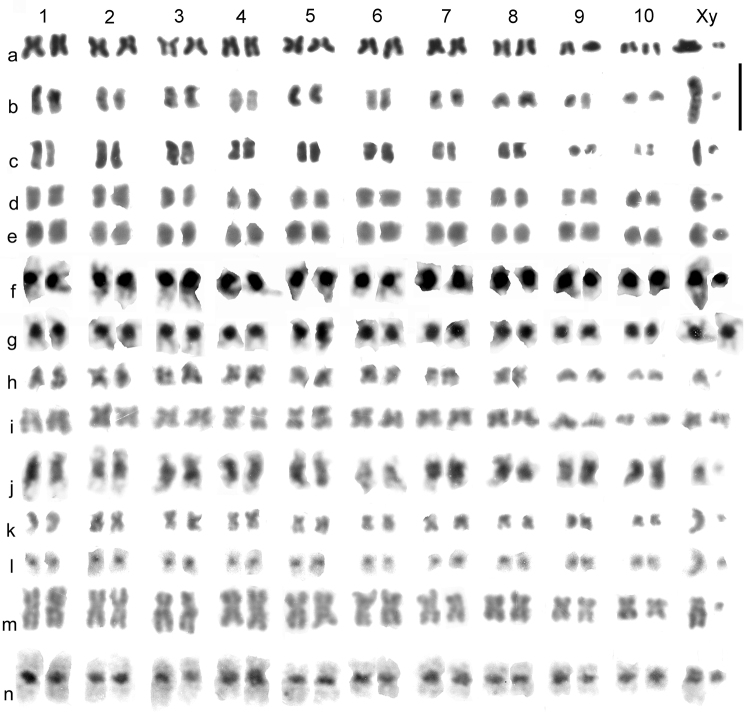
Mitotic chromosomes of Leiodidae, arranged as karyotypes: **a**, **b** *Catops coracinus*, unbanded **a** mid-gut **b** testis **c** *Leiodes calcarata*, testis **d** – **g** *Cantabrogeus luquei*, mid-gut **d**, **e** ♂ unbanded **f** ♂ C-banded **g** ♀ C-banded **h**, **i** *Espanoliella luquei*, mid-gut, unbanded **h** ♂ **i** ♀ **j** *Fresnedaella lucius*, testis, C-banded **k**, **l** *Notidocharis uhagoni*, ♂, mid-gut **k** unbanded **l** the same nucleus C-banded **m**, **n** *Quaestus pasensis*, mid gut **m** unbanded **n** the same nucleus C-banded. Scale bar = 5 µm.

*Catops coracinus*. 2n = 20 + Xy (♂). Giemsa-stained mitotic chromosomes (unbanded), arranged as karyotypes are shown in Fig. 2 a (from mid-gut) and b (from testis). The autosomes are all submetacentric and show an even decrease in length so that pair 10 is about half the length of pair 1. The X chromosome is also submetacentric and is the longest chromosome in the nucleus, about twice the length of autosome 1. The y chromosome is small, almost dot-like. First metaphase of meiosis is shown in Fig. 5f, which shows the 10 autosomal bivalents and the large X chromosome associated with the y in the typical “parachute” association (Xy_p_) of Polyphaga (Smith 1950; Smith and Virkki 1978). Second metaphase of meiosis is shown in Fig. 5g (♂-determining, with a y chromosome) and h (♀-determining, with an X chromosome). The small y and large X (both labeled) are very distinct in these preparations.

*Leiodes calcarata*. 2n = 20 + Xy (♂). Unbanded Giemsa-stained mitotic chromosomes from testis are shown in Fig. 2c. The autosomes and X chromosome are all metacentric or submetacentric. The X chromosome is about the same size as autosome 1, which appears similar in size to that of *Catops coracinus* (compare Fig 1b and c). The autosomes show an even decrease in length from pairs 1 – 8, which pair 8 about half the length of pair 1. There is then an abrupt decrease to pairs 9 and 10, which are about half the length of pair 8. The y chromosome is dot-like. Zygotene of first division of meiosis is shown in Fig. 5a, where the heavily condensed Xy bivalent is distinct. Fig. 5b–d shows first metaphase, with the Xy_p_ bivalent very clear. Fig 5 e shows a second metaphase nucleus, with the y chromosome clearly present.

### Diploid cave-dwelling Leptodirini

**Figure 3.**
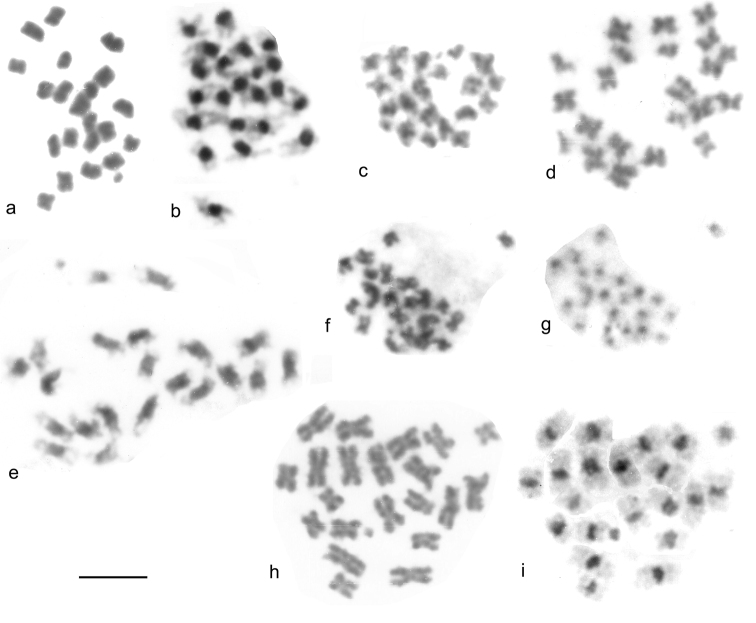
Giemsa stained mitotic chromosomes of Leptodorini as found. **a**, **b** *Cantabrogeus luquei*, mid-gut cell **a** plain **b** C-banded **c**, **d** *Espanoliella luquei*, mid-gut cells, plain **c** ♂ **d** ♀ **e** *Fresnedaella lucius*, testis, C-banded **f**, **g** *Notidocharis uhagoni*, ♂, mid-gut cell **f** plain **g** the same nucleus C-banded **h**, **i** *Quaestus pasensis*, ♂, mid-gut cell **h** plain **i** the same nucleus C-banded. Scale bar = 5 µm.

*Cantabrogeus luquei*. 2n = 20 + Xy (♂), 20 + XX (♀). Unbanded Giemsa-stained mitotic chromosomes from mid-gut cells are shown as karyotypes in Fig 2 d, e (♂), and chromosomes from a C-banded ♂ mid-gut nucleus is shown in Fig. 2f. Fig. 2g shows chromosomes from a C-banded ♀ mid-gut nucleus. The chromosomes shown in Fig. 2 e and f are shown as found in Fig. 3a, b. All the autosomes, and the X chromosome are metacentric with heavy centromeric C-bands. The autosomes show an even decrease in size along the karyotype, with pair 10 about half the length of pair 1. The X chromosome, about 1.5 × the length of autosome 1, is the largest in the nucleus. The y chromosome is a relatively large dot. These preparations are completely consistent with one another and leave no doubt that 20 + Xy or XX is the true diploid number for this species.

*Espanoliella luquei*. 2n = 20 + Xy (♂), 20 + XX (♀). Unbanded Giemsa-stained mitotic chromosomes from mid-gut cells are shown as karyotypes in Fig. 2h (♂) and i (♀), and as found in Fig. 3c, d. No C-banded preparation is available. The chromosomes in these preparations are all rather condensed, but appear either metacentric or submetacentric, with autosome pair 1 distinctly longer than the others, and a gradual decrease in length from pairs 2 – 10. The X chromosome appears similar in length to the medium-sized autosomes and the y chromosome is dot-like. As with *Cantabrogeus luquei*, the preparations are consistent with one another and leave no doubt that the chromosome number reported here is correct.

*Fresnedaella lucius*. 2n = 20 + Xy (♂), 20 + XX (♀). Fig. 2j shows spontaneously C-banded Giemsa-stained mitotic chromosomes from testis. Fig 3e shows these chromosomes as found. The female preparations (not shown) were from mid-gut, completely unbanded and with the chromatids beginning to separate following maximum contraction at metaphase. They are, however, adequate to confirm the chromosome number. Spontaneous C-banding was frequent among testis preparations from these Leptodirini, but in most cases the preparations were not adequate for preparation of karyotypes. The X chromosome is the smallest in the nucleus, apart from the dot-like y. The C-bands are particularly weak in autosome 6 and slightly weaker than most in autosome 2 and the X chromosome, but in the remaining autosomes they are very strong. All the autosomes and the X chromosome are metacentric to submetacentric.

*Notidocharis uhagoni*. 2n = 20 + Xy (♂). Fig. 2k, l shows karyotypes from a ♂ mid-gut cell, unbanded and C-banded. These chromosomes are shown as found in Fig. 3f, g. The autosomes and X chromosome are all metacentric or submetacentric, with small centromeric C-bands. The X chromosome, about twice as long as autosome 1, is the longest in the nucleus, and the autosome lengths decrease fairly evenly along the karyotype, with autosome par 10 about half the length of pair 1. The y chromosome is dot-like. As with the other species, the preparations are consistent and there is no reason to doubt the number obtained.

*Quaestus pasensis*. 2n = 20 + Xy (♂). Fig. 2m, n shows karyotypes from a mid-gut cell, unbanded and C-banded, and Fig. 3h, i shows these chromosomes as found, before and after C-banding. Autosome pair 3 and the X chromosome are more or less subacrocentric while the other autosome pairs are metacentric to submetacentric. The autosome pairs decrease in length evenly along the karyotype, with pair 10 slightly less than half the length of pair 1. The X chromosome is about as long as autosome pair 5 and the y chromosome is dot-like. The centromeric C-bands are distinct, with those of autosome pairs 1 and 4 about twice the size of the others. As with the other species, the preparations obtained are completely consistent with one another and leave no reason to doubt their accuracy.

### Triploid species

**Figure 4.**
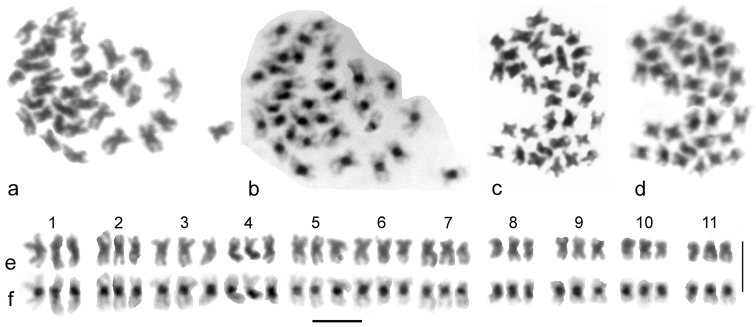
*Cantabrogeus* triploid species, mitotic chromosomes from mid-gut nuclei. **a–d** the chromosomes as found **a**, **c** unbanded **b**, **d** the same nuclei C-banded **e**, **f** karyotypes assembled from the nucleus figured in **a** & **b**. Scale bar = 5 µm.

*Cantabrogeus* sp. (Salgado et al. in press) 3n = 33 (♀). Fig. 4 shows mid-gut chromosomes of this species. Two nuclei are shown, both unbanded (Giemsa-stained) and C-banded. Unbanded and C-banded karyotypes from the nucleus shown in Fig. 4a and b are shown as Fig. 4e and f. As only females are present it is not possible to identify the X chromosome, but the results are totally consistent with a triploid number and a haploid complement of 10 + X. Pairs 7 and 11 are subacrocentric, pairs 3, 5, 8 and 10 are clearly submetacentric, and the remainder are more or less metacentric. The chromosome lengths decrease rather evenly along the karyotype, with pair 11 rather more than half the length of pair 1. The centromeric C-bands are bold and distinct, but slightly smaller than those of *Cantabrogeus luquei*. There is no chromosome of this species which invites obvious comparison with the X chromosome of *Cantabrogeus luquei*, so there is no hint in this material about which is the X chromosome.

**Figure 5.**
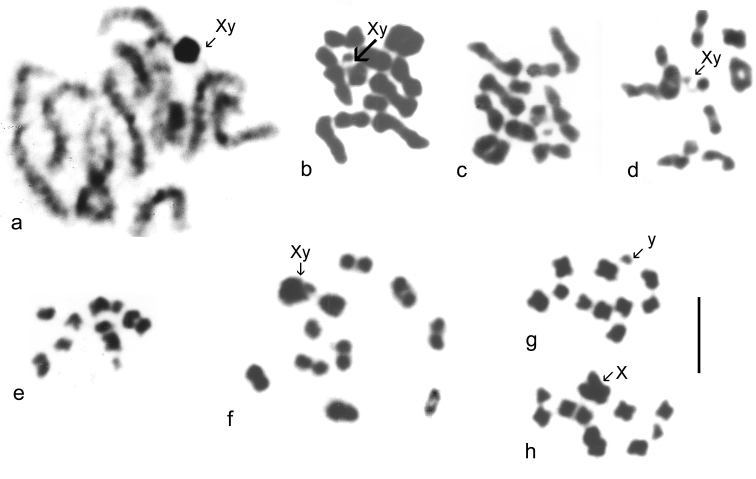
Meiosis of Leiodidae (surface forms). **a–e** *Leiodes calcarata* **f–h** *Catops coracinus* **a** prophase I, zygotene **b–d** metaphase I **e**, metaphase II, ♂-determining, with y chromosome **f** metaphase I **g**, **h** metaphase II **g** ♂-determining, with y chromosome **h** ♀-determining, with X chromosome. Scale bar = 5 µm.

### Phylogenetic placement of the triploid species.

**Figure 6.**
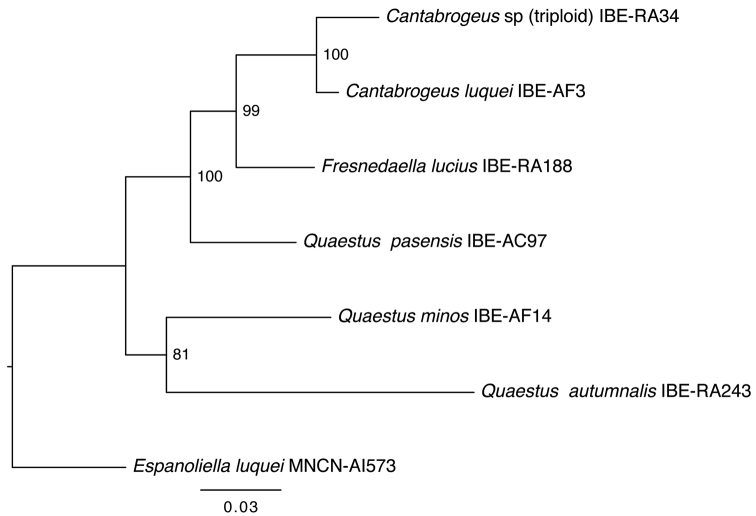
Phylogenetic relationships of triploid *Cantabrogeus* sp., obtained with the same genes and methodology used by Ribera et al. (2010). Courtesy of I. Ribera, Institute of Evolutionary Biology, CSIC–UPF (Barcelona, Spain).

DNA from a specimen captured on June 6, 2009 (ref. IBE-RA34) was analyzed by I. Ribera (Salgado et al. in press), using the genetic methods described in Ribera et al. (2010) and used to establish the phylogenetic relationships of the new triploid species. Cladistic analysis of the sequences identified the sister relationship between the triploid *Cantabrogeus* sp. and other supraflagellates of the ‘*Quaestus*’ series (Fig. 6). The analysis of the obtained sequences shows that *Cantabrogeus* is the sister group of *Fresnedaella lucius* and *Quaestus pasensis* and this clade, in turn, is the sister group of *Quaestus minos* and *Quaestus autumnalis* (Salgado et al. 2011). Moreover, the whole clade has a sister relationship with the genus *Espanoliella*. All clades receive high support values. The sister group relationship between *Cantabrogeus*, *Fresnedaella* and *Quaestus pasensis* is recovered in 99-100% of the bootstrap replicates of RAxML (Fig. 6).

## Discussion

**Figure 7.**
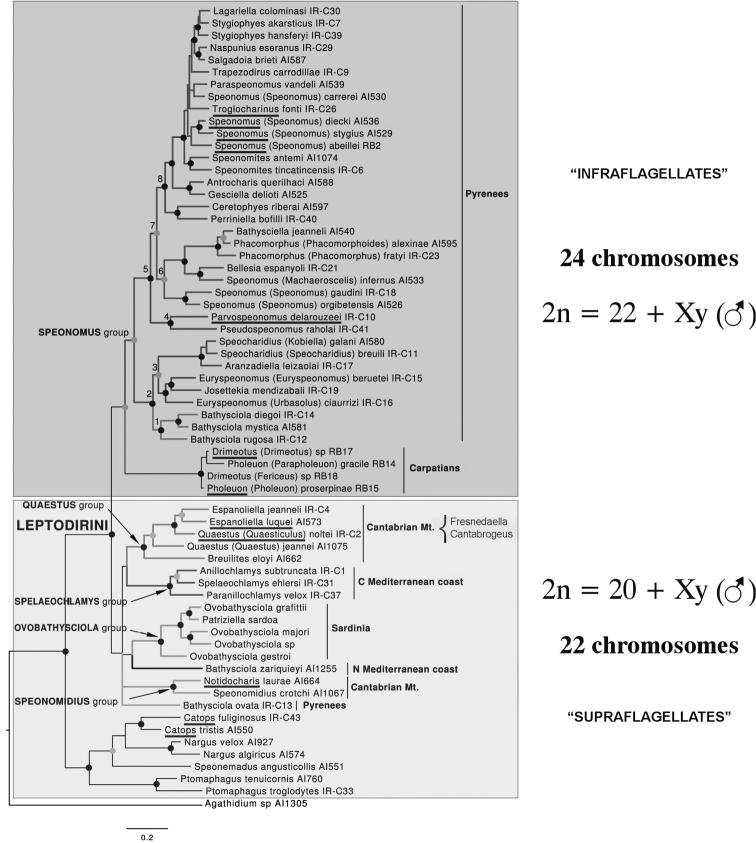
Phylogram obtained from Ribera et al. (2010), modified to show the reconstructed evolution of the chromosome number. Genera whose chromosome complements are known are underlined.

As mentioned in the Introduction, the results obtained here appear both surprising and puzzling as all published data indicate a diploid chromosome number of 22 + Xy (♂), which Buzila and Marec (2000) considered to be conservative for the family. The 10 species involved, with their localities of origin and map numbers, are listed in Table 2. This shows that all these species belong in most derived section of the Leptodirini, the infraflagellates of Jeannel (1955) characterised by the basal region of the internal sac of the aedeagus having a Y-shaped ventral sclerite, the Y-piece. Fresneda et al. (2007), in their cladistic analysis of leiodid morphology, concluded that the infraflagellates were a monophyletic group but that the other of Jeannel’s groups, the supraflagellates, characterised by having a dorsal flagellum in the basal region of the internal sac of the aedeagus, was a paraphyletic assemblage of less highly derived species. This arrangement is supported and amplified by the DNA work of Ribera et al. (2010). Moreover, Salgado et al. (2011) published an article in which the phylogenetic relationships of *Fresnedaella* and *Cantabrogeus*, and its allied taxa were mentioned. These authors show that, while all the species previously studied chromosomally, including those from the Carpathians, belong to the derived infraflagellates, those reported here all belong in the more basal supraflagellate assemblage (Figs 6, 7). It therefore seems that the more primitive Leiodidae have a chromosome complement comprising 10 pairs of autosomes and Xy/XX sex chromosomes – the only exception being one triploid species of *Cantabrogeus*. So, far from being the conservative chromosome number for Leiodidae, 11 pairs plus Xy/XX sex chromosomes is a derived feature of the advanced infraflagellates. It is also worth noting that if Smith’s (1950) suggestion that the ancestral Polyphagan chromosome complement was 9 pairs + Xy/XX is correct, then the basic number for Leiodidae involves an increase of one pair of autosomes, while the advanced infraflagellates show an additional increase of one pair.

**Table 2.** Species of Leptodorini for which chromosome details have been published: species with map No. As shown in Fig. 1, area from which the material was collected, and publication reference.

**Species (Map No.)**	**Area**	**Reference**
*Speonomus hydrophilus* (Jeannel, 1908) (No. 9)	Pyrenees, France-Spain	Durand and Juberthie-Jupeau 1980
*Speonomus pyrenaeus* (Lespès, 1857) (No.10)		
*Parvospeonomus delarouzeei* (Fairmaire, 1860) (No.11)		Alegre and Escolà 1983
*Troglocharinuselongatus* Zariquiey, 1950(=*variabilis* Bellés, 1978)(No. 12)		
*Troglocharinuselongatus jacasi* (Lagar, 1966) (No. 13)		
*Troglocharinuselongatus schibii* (Español, 1972) (No. 14)		
*Troglocharinuselongatus ferreri* (Reitter, 1908) subspecies *pallaresi* Bellés, 1973 (No. 15)		
*Troglocharinuselongatus kiesenwetteri* (Dieck, 1869) (No. 16)		
*Pholeuon knirschi* Breit, 1911 (No. 17)	Carpathians, Romania	Buzila and Marec 2000
*Drimeotus kovacsi* Miller, 1856 subspecies *viehmanni* Ieniştea, 1955 (No. 18)
